# *LRIG1* expression and colorectal cancer prognosis

**DOI:** 10.1186/s12920-020-00846-2

**Published:** 2021-01-18

**Authors:** Maryam Bakherad, Mahdieh Salimi, Seyed Abdolhamid Angaji, Frouzandeh Mahjoubi, Tayebeh Majidizadeh

**Affiliations:** 1 Department of Cell and Molecular Biology, Faculty of Biological Sciences, Kharazmi University, Tehran, Iran; 2 Department of Medical Biotechnology, National Institute of Genetic Engineering and Biotechnology (NIGEB), Tehran, Iran

**Keywords:** Prognostic indicator, Colorectal cancer, CRC, *LRIG1*, Gene expression

## Abstract

**Background:**

To make the right treatment decisions about colorectal cancer (CRC) patients reliable predictive and prognostic data are needed. However, in many cases this data is not enough. Some studies suggest that *LRIG1* gene (*leucine-rich repeats and immunoglobulin-like domains1*) has prognostic implications in different kinds of cancers.

**Methods:**

One hundred and two patients with colorectal cancer were retrospectively analyzed for LRIG1 expression at both mRNA and protein levels. SYBR Green Real-Time RT-PCR technique was used for mRNA expression analyses and *Glyceraldehyde-3-Phosphate Dehydrogenase* gene (*GAPDH*) was considered as a reference gene for data normalization. LRIG1 protein expression was analyzed using Immunohistochemistry. Additionally, appropriate statistic analyses were used to assess the expression of *LRIG1* in test and control groups. The prognostic significance of LRIG1 expression was analyzed using the univariate and multivariate analyses.

**Results:**

The data revealed that the expression of *LRIG1* in both mRNA and protein levels was down regulated in colorectal tumor tissues (*P* < 0.01) but is not clinically relevant prognostic indicator in CRC.

**Conclusions:**

Therefore, it is suggested that *LRIG1* expression analyses may not be considered as an important issue when making informed and individualized clinical decisions regarding the management of colorectal cancer patients.

## Background

Colorectal Cancer (CRC) is considered as one of the most significant cancers worldwide, with about 1 million new cases and more than 550,000 deaths per year [1]. Despite the significant advances in the diagnosis of CRC, the survival rate decreases for patients diagnosed with metastatic and regional disease [2]. The reported overall median survival time of CRC is only 1.1 years [3]; therefore, understanding about biological factors with impact on CRC is very important.

The lack of predictive and prognostic biomarkers with the ability to predict therapy response and recurrence of the disease is an important issue that needs to be addressed. The *LRIG1* (*the leucine-rich repeats and immunoglobulin-like domains 1*) as an emerging tumor suppressor and its paralogs *LRIG2* (*the leucine-rich repeats and immunoglobulin-like domains 2*) and *LRIG3* (*the leucine-rich repeats and immunoglobulin-like domains 3*), are considered to have prognostic significance in various kinds of cancers, such as head-and-neck [4, 5], prostate [6], breast [7, 8], uterine cervical cancer [9–11], and cutaneous squamous cell carcinoma [12], and glioma [13, 14].

The locus of *LRIG1* is located on chromosome 3p14.3. The encoding protein is a transmembrane protein consisting of an extracellular region including three immunoglobulin (Ig)-like domains and fifteen leucine-rich repeats. The leucine-rich repeats and immunoglobulin-like domains have interactions with all four extracellular region binding protein B receptor family members leading to the regulation of receptor levels by subsequent lysosomal degradation and increasing ubiquitination, independent of ligands [15–17]. In addition the LRIG1 is considered as a marker of human epithelial stem cells in a quiescent non-proliferative state [18]. The enhanced proliferation associated with epithelial hyper-proliferation in vivo and stem cell expansion in vitro is the result of the genetic erosion of the leucine-rich repeats and immunoglobulin-like domains [18, 19]. It is recommended by lineage tracing that the leucine-rich repeats and immunoglobulin-like domains mark non-cycling, long-lived stem cells of the ‏4 quiescent intestinal stem cell niche in the crypt [20]; and also progenitor cells in the stomach that are involved in restoring gastric cell mucosa after DMP-777 induced acute damage [21].

Although the LRIG1 plays vital role in cancers, little is known about its association with clinico-patho-physiology characteristics of CRC patients. Here the *LRIG1* expression in the lesions of CRC patients was studied in order to evaluate its relationship with the major clinicohistological predictive factors and its respective impacts on patient prognosis hoping to improve the approaches for colorectal cancer management. Therefore, the main goal of the present study was to compare and analyze the expression levels of the *LRIG1* gene in samples of tumor and normal colorectal tissues of CRC patients by quantitative real-time RT-PCR and immunohistochemical (IHC) techniques. Moreover, to estimate the prognostic indicator of the mentioned gene expression levels, we surveyed their correlations with clinicopathological parameters, as well as the overall survival (OS) of patients with CRC.

## Methods

### Patient information

A total of 102 cases of colorectal cancer from Imam Khomeini Hospital, Tehran, Iran were selected. The average age was 55.0 ± 10.0 years. The inclusion criteria were post-operative diagnosis of primary CRC based on histopathology. The study was approved by the Ethics Committee of the NIGEB based on the Helsinki declaration. All the patients signed informed consent. The ethics code number is IR.NIGEB.1395.11.10.E. The patients' characteristics are presented in Table 1. Overall survival (OS) was defined as the time from the date of primary treatment to the date of death from any cause or until the date of the last follow-up. Disease-free survival (DFS) for patients with CRC was defined as the time from the date of primary treatment to the date of diagnosis for recurrence or disease or to the date of the last follow-up.

**Table 1:** Baseline characteristics of colorectal cancer patients

Characteristic	Number (%)
Number of patients	102 (100)
Gender	
Male	49 (48)
Female	53 (52)
Age (years, mean ± SD)	55.00 ± 10
Pathological stage	
Stage 1	27 (26)
Stage 2	29 (28)
Stage 3	24 (24)
Stage 4	22 (22)
Tumor size	
< 5 cm	47 (46)
5–8 cm	31 (30)
8–10 cm	14 (14)
≥ 10 cm	10 (10)
Lymph nodes metastasis	
Positive	49 (48)
Negative	53 (52)
Other metastasis	22 (22)

### Immunohistochemical analysis

**Fig. 1:**
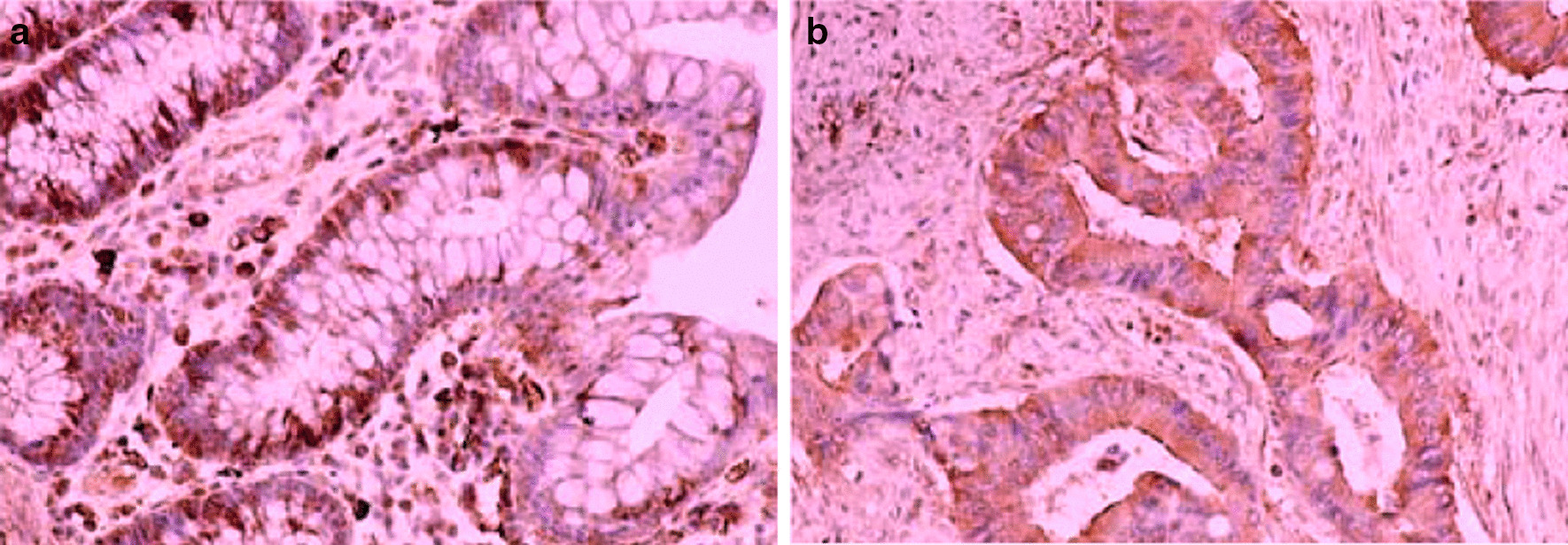
Expression of LRIG1 protein in colorectal cancer (CRC) and unchanged colon mucosa as assessed by immunohistochemistry. **a** Section of unchanged colon mucosa and **b** CRC show the immunoreactivity. Magnification, × 100

Surgical specimens were formaldehyde fixed paraffin embedded and sectioned at a thickness of 4 μm followed by xylene dewaxing, ethanol gradient rehydration and harnessed to high pressure and temperature for antigen retrieval. The slices were incubated in H_2_O_2_ harnessed to the primary antibody, rinsed with phosphate buffered saline (PBS), then harnessed to secondary and mouse anti-human LRIG1 monoclonal antibody, respectively. The slices incubated with PBS instead of the primary antibody were used as the negative control. The sections were assessed using an Olympus BX41 light microscope (Olympus, Tokyo, Japan) by a pathologist. The scale based on the reaction intensity were used to assess immunoreactivity in enterocytes or cancer cells of the studied sections (0, no reaction; 10, up to 10%; 30, 11–30%; 60, 31–60%; 80, 61–80%; and 100, > 80%) (Fig. 1).

### RNA extraction, cDNA synthesis and LRIG1 mRNA expression analysis

Total RNA was isolated from the colorectal tissue using YTzol kit (Yekta Tajhiz Azma Co, Tehran, Iran) according to the manufacturer’s protocol. cDNA was synthesized following the manufacturer’s instructions (Cinaclon Co, Tehran, Iran) and stored at − 20 °C until analyzed. The primer sequences for *glyceraldehyde-3-phosphate dehydrogenase* (*GAPDH*) and *LRIG1* genes were designed using primer 3 software (https://primer3.ut.ee/) and then blasted using https://www.ncbi.nlm.nih.gov/tools/primer-blast/. The designed primer sequences are shown in Table 2. The Real-time RT-PCR amplifications were conducted in a final volume of 15 μl reaction mixture containing 1 μl of cDNA, 7.5 μl RealQ plus 2 × master mix green (Ampliqon, Denmark), 0.6 μl (10 μmol/l) of each primer and 5.3 μl sterilized water, using the Rotor-Gene Q System (QIAGEN Hilden, Germany). The cycling conditions were as follows: 15 min at 95 °C followed by 40 cycles of denaturation at 95 °C for 30 s, 60 °C for 30 s and 72 °C for 30 s for the *LRIG1* and also the *GAPDH*, which was used as a normalizer. Experiments were performed in triplicates for each data point. The linear standard curve (from 0.1 to 1000 ng) assessed by ultraviolet spectrophotometer was used for amplification efficiency determination of each primer pair. The standard curves showed good linearity and amplification (100%). The data was presented as the fold change in gene expression normalized to an endogenous reference gene relative to the controls using 2^−ΔΔCT^ method.

**Table 2:** The primers for real-time quantitative reverse transcription PCR

Gene name	Primer sequence	Product size (bp)	Annealing temperature (°C)
*LRIG1*	F: CTGCATGAGTTGGTCCTGTCC	112	60
	R: TGTGGCTGATGGAATTGTGG		
*GAPDH*	F:GCAGGGGGGAGCCAAAAGGGT	219	60
	R: TGGGTGGCAGTGATGGCATGG		

*LRIG1* leucine-rich repeats and immunoglobulin-like domain-1, *GAPDH* Glyceraldehyde-3-phosphate dehydrogenase

### Statistical analysis

Graphpad Prism 8.0.2 (California Corporation, USA) and SAS computer software version 9.1 (SAS Institute Inc., Cary, NC, USA) were used to analyze the data. The Mann–Whitney U test and Kruskal–Wallis test were performed for numerical data and the Chi-square test was used to analyze the relationship between parameter data. Numerical data are presented as the mean ± standard deviation (SD). Differences were considered as statistically significant if *P* < 0.05. The Cox proportional-hazards model was used for univariate and multivariate analyses to identify the independent prognostic factors for OS, DFS.

## Results

### LRIG1 expression and clinicopathological features

#### *LRIG1* mRNA expression

**Fig. 2:**
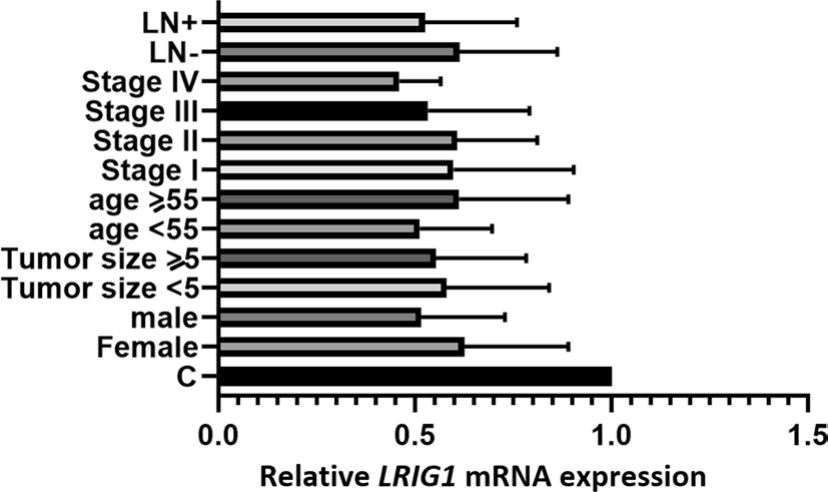
Evaluation of the *LRIG1* relative mRNA expression in tumor tissues based on clinopathologic situations. *LN* lymph node metastasis, *C* normal control

The *LRIG1* mRNA expression was significantly down-regulated in Colorectal cancerous tissues compared with normal control (*P* < 0.01). The mean of *LRIG1* relative expression in cancerous tissues compared with normal control was 0.57 ± 0.24 with a range of 0.23 to 1.2. About 40% of cancerous samples showed the relative expression < 0.5 that was considered as down-regulation. As shown in Fig. 2, there were no significant differences between different demographical and clinicopatological characteristics of CRC patients and *LRIG1* expression (*P* > 0.05).

#### LRIG1 protein expression

The LRIG1 immunoreactivity was found in the cytoplasm of enterocytes as well as cancer cells of the analyzed tissues (Fig. 1).

The tumor LRIG1-positive rate in colorectal cancer tumors was 37.4%, which was significantly lower than that in control tissues (57.7%, *P* < 0.05). There was no significant correlation in the expression of the LRIG1 with tumor size, tumor diameter, tumor differentiation, age, and the number of positive mesenteric lymph nodes or vascular cancer embolus (*P* > 0.05) (Table 3).

**Table 3:** Clinicopathological variables and their correlation with immunohistochemical expression of LRIG1 in primary tumors

Clinicopathological variables	CRC patients n (%)	Score < 30 n (%)	Score ≥ 30 n (%)	*P* value
Age				
≤ 57	49 (48)	25 (51.0)	24 (49.0)	0.451
> 57	53 (52)	26 (49.1)	27 (50.9)	
Primary tumor location				
Colon	73 (72)	33 (45.2)	40 (54.8)	0.321
Rectum	29 (28)	14 (48.3)	15 (51.7)	
Differentiation				
High	12 (12)	5 (42.9)	7 (57.1)	0.461
Mid-low	88 (86)	39 (44.3)	49 (55.7)	
Unknown	2 (2)	1 (50.0)	1 (50.0)	
Positive lymph node	49 (48)	26 (53.6)	23 (46.4)	0.243
Tumor diameters				
< 5 cm	47 (46)	22 (46.8)	25 (53.2)	0.763
5–8 cm	31 (30)	15 (48.4)	16 (51.6)	
8–10 cm	14 (14)	6 (42.2)	8 (57.8)	
≥ 10 cm	10 (10)	4 (37.2)	6 (62.8)	
Vascular cancer embolus				
Yes	65 (64)	34 (53.2)	31 (46.8)	0.382
No	37 (36)	19 (51.3)	18 (48.7)	

### Univariate and multivariable analyses of survival impact of LRIG1 expression in patients with CRC

The univariate and multivariate analyses were performed to investigate independent prognostic factors for Overall survival (OS) and Disease-free survival (DFS) in patients with colorectal cancer using the Cox proportional-hazards model (Table 4). The analysis results demonstrated no significant difference for parameters in terms of OS (*P* > 0.05) and DFS (*P* > 0.05).

**Table 4:** Univariate and multivariable analysis of prognostic indicators on overall survival and disease-free survival for the prognostic significance of LRIG1 expression of colorectal cancer patients (N = 102)

Parameters	Overall survival				Disease-free survival			
	Univariate analysis		Multivariable analysis		Univariate analysis		Multivariable analysis	
	HR (95% CI)	*P* value	HR (95% CI)	*P* value	HR (95% CI)	*P* value	HR (95% CI)	*P* value
Age (years)	0.698 (0.376–1.271)	0.243	0.689 (0.303–1.515)	0.354	0.830 (0.453–1.518)	0.555	0.981 (0.439–2.231)	0.991
(≥ 57 vs < 57)								
Gender	0.944 (0.525–1.658)	0.953	0.859 (0.409–1.755)	0.667	1.029 (0.574–1.806)	0.962	0.923 (0.433–1.921)	0.819
(Male vs female)								
Location	0.833 (0.454–1.495)	0.534	0.996 (0.449–2.164)	0.982	0.775 (0.421–1.388)	0.389	1.044 (0.477–2.237)	0.946
Rectum versus colon								
Tumor size	0.887 (0.490–1.643)	0.736	1.099 (0.512–2.310)	0.837	0.887 (0.489–1.641)	0.733	1.054 (0.471–2.311)	0.928
(≥ 5 cm vs < 5 cm)								
LN metastasis	1.073 (0.588–1.923)	0.855	0.998 (0.448–2.181)	0.581	1.091 (0.604–1.965)	0.787	0.824 (0.364–1.817)	0.625
Yes versus no								
Vascular invasion	1.694 (0.921–2.932)	0.101	1.171 (0.519–2.629)	0.718	1.566 (0.869–2.782)	0.148	1.289 (0.549–3.068)	0.564
Yes versus no								
LRIG1 expression	1.252 (0.690–2.260)	0.473	1.826 (0.823–4.004)	0.151	1.077 (0.597–1.904)	0.831	1.451 (0.656–.081)	0.271
Positive versus negative								

## Discussion

Cancer is one of the most important and prevalent diseases with poor prognosis and there is no effective method to treat and predict the procedure of tumorigenesis. Finding the appropriate biomarkers of cancer prediction or prognosis will have huge importance in cancer management. Nowadays the new strategies searching for informative biomarkers in cancer management have attracted good attention in the world. The increasing evidences have demonstrated the leucine-rich repeats and immunoglobulin-like domain as an independent prognosis factor and predictive biomarker of clinicopathology in variety of tumors. Due to inconsistency on the effect of the LRIG1 in different types of tumors, the present study was carried out to investigate the prognostic importance of the LRIG1 expression and its relationship with clinicopathological significance in CRC. In the present study, the expression of LRIG1 at both mRNA and protein levels was significantly decreased in CRC tumors compared with normal control but, the high levels of leucine-rich repeats and immunoglobulin-like domains expression were not significantly associated with longer overall survival, which was consistent with the conclusion of subgroup analysis. These results suggested that the LRIG1 was not a prognostic marker in CRC tumors. Meanwhile, the LRIG1 expression was significantly lower in cancer tissues than normal ones and the same result was detected with no heterogeneity in subgroup analysis based on the type of tumor. The higher levels of LRIG1 expression was not also related to positive HPV status and tumor progression assessed by its association with degree of differentiation. Also, there was no association between the LRIG1 expression and lymphatic metastasis. Some genes, such as *K-ras* and epidermal growth factor receptor (*EGFR*) and human epidermal growth factor receptor 2 (*HER2*) are reported to be involved in the progress and development of colorectal cancer [22–25], but the *LRIG1* roles in colorectal cancer have not been well studied and remained contradictory.

Some studies showed that distal and proximal colon cancers differ in terms of molecular, pathological, and clinical features [26, 27]. The present data revealed that there was not any correlation between LRIG1 expression and bilateral and peritoneal CRC metastasis (*P* > 0.05) and also with age, synchronous or metachronous CRC or primary tumor location (*P* > 0.05). Although, earlier studies proposed that LRIG1 expression was associated with a good prognosis in terms of overall survival (OS) and might act as a predictive factor for characteristics of cancer patients [28], whether the LRIG1 expression could predict a lower risk of CRC remains doubtful.

## Conclusions

In conclusion, our studies revealed that although *LRIG1* was down regulated in CRC and primary tumors of CRC patients but, its expression in both mRNA and protein levels, was not clinically relevant prognostic indicator in CRC. Therefore, it is suggested that *LRIG1* expression analyses may not be important when making informed and individualized clinical decisions regarding the management of colorectal cancer patients.

## Data Availability

The datasets used and/or analyzed during the current study are available from the corresponding author on reasonable request.

## Abbreviations

CRC: Colorectal cancer
GAPDH: Glyceraldehyde-3-phosphate dehydrogenase
LRIG1: Leucine-rich repeats and immunoglobulin-like domains 1
Ig: Immunoglobulin
PBS: Phosphate buffered saline
OS: Overall survival
DFS: Disease-free survival
PCR: Polymerase chain reaction
